# SLC30A9: an evolutionarily conserved mitochondrial zinc transporter essential for mammalian early embryonic development

**DOI:** 10.1007/s00018-024-05377-y

**Published:** 2024-08-19

**Authors:** Jing Ge, Huihui Li, Xin Liang, Bing Zhou

**Affiliations:** 1 School of Life Sciences, Tsinghua University, Beijing, 100084 China; 2 Shenzhen Key Laboratory of Synthetic Genomics, Guangdong Provincial Key Laboratory of Synthetic Genomics, Key Laboratory of Quantitative Synthetic Biology, Shenzhen Institute of Synthetic Biology, Shenzhen Institute of Advanced Technology, Chinese Academy of Sciences, Shenzhen, 518055 China; 3 Faculty of Synthetic Biology, Shenzhen University of Advanced Technology, Shenzhen, 518055 China

**Keywords:** CG8632, ZnT49B, ZnT9, GH/IGF, Electron transport chain

## Abstract

SLC30A9 (ZnT9) is a mitochondria-resident zinc transporter. Mutations in SLC30A9 have been reported in human patients with a novel cerebro-renal syndrome. Here, we show that ZnT9 is an evolutionarily highly conserved protein, with many regions extremely preserved among evolutionarily distant organisms. In *Drosophila melanogaster* (the fly), *ZnT9* (*ZnT49B*) knockdown results in acutely impaired movement and drastic mitochondrial deformation. Severe *Drosophila ZnT9 (dZnT9)* reduction and *ZnT9*-null mutant flies are pupal lethal. The phenotype of *dZnT9* knockdown can be partially rescued by mouse ZnT9 expression or zinc chelator TPEN, indicating the defect of *dZnT9* loss is indeed a result of zinc dyshomeostasis. Interestingly, in the mouse, germline loss of *Znt9* produces even more extreme phenotypes: the mutant embryos exhibit midgestational lethality with severe development abnormalities. Targeted mutagenesis of *Znt9* in the mouse brain leads to serious dwarfism and physical incapacitation, followed by death shortly. Strikingly, the GH/IGF-1 signals are almost non-existent in these tissue-specific knockout mice, consistent with the medical finding in some human patients with severe mitochondrial deficiecny. *ZnT9* mutations cause mitochondrial zinc dyshomeostasis, and we demonstrate mechanistically that mitochondrial zinc elevation quickly and potently inhibits the activities of respiration complexes. These results reveal the critical role of ZnT9 and mitochondrial zinc homeostasis in mammalian development. Based on our functional analyses, we finally discussed the possible nature of the so far identified human SLC30A9 mutations.

**Supplementary Information:**

The online version contains supplementary material available at 10.1007/s00018-024-05377-y.

## Significance statement

ZnT is a family of proteins usually involved in zinc transport from the cytosol to intracellular organelles or extracellular area. One member of the ZnT family, ZnT9, participates in mitochondrial zinc transport. ZnT9 mutations in humans cause brain and kidney defects. Here, we show that *ZnT9* mutation in the fruit fly is pupae-lethal and, in the mouse, results in embryonic disappearance. Brain-specific disruption of *Znt9* in the mouse causes movement disorder, loss of growth hormone, and development abnormality. We further indicate that ZnT9 moves zinc out of mitochondrion and its loss leads to zinc accumulation in the mitochondrion. Zinc accumulation impairs mitochondrial respirational activity. The data shed light on the function of ZnT9 in development, especially that of mammals. The knowledge helps our understating of the corresponding disease and may even benefit the disease treatment.

**Supplementary Information:**

The online version contains supplementary material available at 10.1007/s00018-024-05377-y.

## Introduction

Zinc is involved in a large number of biological processes and functions as catalytic, structural and signaling components [1, 2]. Zinc deficiency and extreme excess may both lead to multifarious diseases. Zinc deficiency can result in disrupted immune system, gastrointestinal dysfunction, endocrine dyscrasia, neurologic defects, and is connected to cancer, accelerated aging, degenerative disorder and cardiovascular diseases [3, 4]. Excess zinc can damage neurons and cause neuronal death through oxidative stress and reduced neuronal energy production [5, 6]. Thus, maintenance of zinc homeostasis is of great importance.

Zinc cannot permeate across the biological membrane. Zinc homeostasis is tightly controlled by zinc transporters including ZIP family (SLC39A) and ZnT family (SLC30A) [7]. There are 14 proteins in ZIP family and 10 proteins in ZnT family in mammals. ZIP proteins mostly transport zinc from outside of cells or intracellular organelles into the cytosol, and ZnT proteins usually facilitate the mobilization of zinc from the cytosol to extracellular area or intracellular compartments [8]. Therefore, ZnT proteins help in providing zinc to organellar proteins for normal functions or zinc storage, and alternatively, they can move zinc to the extracellular area to prevent zinc toxicity [9]. Intracellular zinc homeostasis is controlled in a concerted manner by zinc trafficking as mediated by ZIPs/ZnTs, and by metallothioneins (MTs), which contain 20 cysteines and bind zinc with high affinities [10]. MTs are in addition regulated by MTF-1 (metal-responsive transcription factor-1), a transcription factor owning zinc finger domains [11]. MTF-1 binds to the MRE (metal responsive element) in the enhancer regions of *MTs* once it combines with zinc [12].

Besides many physiological processes in the cytosol, zinc also affects actions in the mitochondrion, such as the tricarboxylic acid (TCA) cycle and the electron transport chain (ETC) activities; it is known that under the case of excessive zinc the components of ETC, glycolysis and TCA cycle are impaired [13–16]. Thus, the maintenance of mitochondrial zinc homeostasis is also critical. In this regard and during the execution of this project, it was reported that ZnT9 is a mitochondrial zinc transporter in *Caenorhabditis elegans* and HeLa cells, transporting zinc from mitochondria to the cytosol [17–19]. Deletion of *ZnT9* caused zinc accumulation in mitochondria and impaired the function of mitochondria [17–19]. Deng et al. also found that loss of *ZnT9* in *C. elegans* resulted in viable worms with swollen mitochondria, impaired fertility, and defective sperm activation [17]. However, at the systemic level, the role of ZnT9 in other organisms, in particular the mammal, remains elusive.

A few years ago, it was first reported that SLC30A9 (ZnT9) mutations in humans cause a novel cerebro-renal syndrome called Birk–Landau–Perez Syndrome [20]. The clinical features of this disease are intellectual disability, dystonia, movement disorder, oculomotor apraxia, and renal abnormality. More cases of the disease were found recently [21–23]. Although the genetic basis of this disease is revealed, its etiology is not clarified, and further investigations are needed. In this study, we demonstrated that ZnT9 is evolutionarily very conserved. We first analyzed *ZnT9* disruption in the fly and found it is essential for viability. We proceeded to the rodent study and discovered that ZnT9 is far more important in mammals than in the worm. It is a potent regulator of GH/IGF-1 signaling and affects many other signaling processes but not dopamine formation. The present findings revealed the importance of ZnT9 in controlling mitochondrial zinc homeostasis and could help to better understand the pathogenesis of Birk–Landau–Perez Syndrome.

## Results

### ZnT9 is an evolutionarily conserved zinc transporter with a mitochondrial presequence

**Fig. 1:**
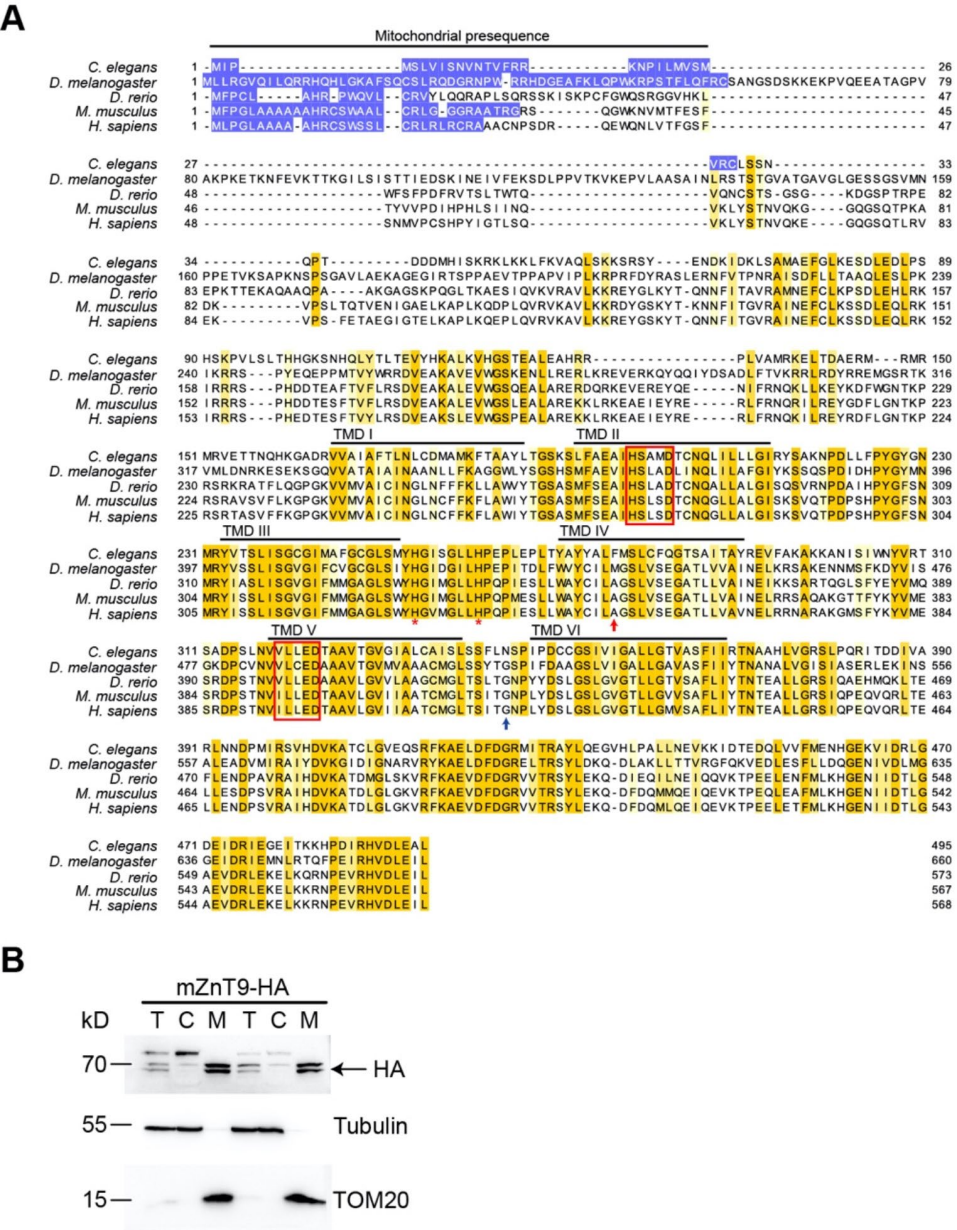
SLC30A9 (ZnT9) is an evolutionarily conserved mitochondrion-targeted zinc transporter. (**A**) Shown here is a comparison of ZnT9 from *C. elegans*, *D. melanogaster*, *D. rerio*, *M. musculus* and *H. sapiens*. Potential mitochondrial signals are colored by purple. Highly conserved residues are colored by yellow. Mutations in human SLC30A9 (pAla350del and p.Gly418Val, marked with a red and blue arrow, respectively) causing a novel cerebro-renal syndrome were previously reported [20, 23]. The six potential transmembrane domains are labelled above with lines. Notably, ZnT9 contains a set of highly conserved regions outside of the transmembrane domains. Also, two highly conserved His residues, indicated with * in the figure, exist between TM III and TM IV. Another distinctive feature is that the motif HXXXD occurring in TM V of most ZnTs is changed to V/IXXXD in ZnT9. This HXXXD motif, together with another HXXXD sequence in TM II, are believed to coordinate for zinc-binding in ZnTs. Accession numbers for depicted sequences are as follows: *C. elegans* ZnT9, NP_497603.3; *D. melanogaster* ZnT9, NP_725207.1; *D. rerio* ZnT9, NP_001008575.1; *M. musculus* ZnT9, NP_848766.2; *H. sapiens* ZnT9, NP_006336.3. (**B**) mZnT9 localizes to the mitochondrion. mZnT9-HA was expressed in 4T-1 (mouse breast cancer cell line) cells. Mitochondria were isolated and were marked by TOM20. The expression of mZnT9-HA was examined by western blotting using anti-HA antibody. T: total cell; C: cytosol; M: mitochondria

There are ten ZnTs in mammals, but in *D. melanogaster*, only seven. They, thus, do not all correspond to each other. Previous phylogenetic analyses between the flies and humans showed that several ZnTs have clear counterparts (orthologues); they are notably ZnT1, ZnT7 and ZnT9 [24]. Alignment of presumed ZnT9 orthologues from *C. elegans*, *D. melanogaster*, *D. rerio*, *M. musculus* and *H. sapiens* revealed that ZnT9 is highly conserved evolutionarily (Fig. 1A). All of them contain a potential mitochondrial presequence, suggesting the mitochondrial localization. The mitochondrial presence of the *C. elegans* ZnT9 and human ZnT9 has indeed been established [17–19]. Since the previous publications used GFP fusion to localize ZnT9 to the mitochondrion, to minimize the tag effect, we used the small tag HA to attach to mZnT9 to confirm its mitochondrial residence. mZnT9-HA was expressed in 4T-1 (mouse breast cancer cell line) cells. Mitochondria were isolated and expression of mZnT9-HA was examined by western blotting using anti-HA antibody. The results indicated that mZnT9-HA is predominantly in the mitochondrial fraction and very little in the post-mitochondrial fraction (Fig. 1B), consistent with previous reports.

As anticipated, there are six putative transmembrane domains (TM) in ZnT9, as in other ZnT proteins [25]. Unsurprisingly, the six putative transmembrane domains are highly conserved during evolution. Within the region between TM III and TM IV of ZnT9, two conserved histidine residues notably exist (labelled with * in Fig. 1A). Another outstanding feature is the motif V/IXXXD (X stands for any amino acid) in TM V of ZnT9, which is HXXXD in many other ZnTs and considered to bind zinc in coordination with the HXXXD motif in TM II [26], which is preserved in ZnT9. Besides the transmembrane domains, the C terminus of ZnT9 and some inter-transmembrane regions also exhibit highly conserved blocks, implicating important, albeit unknown biological functions.

### *ZnT9* suppression results in mitochondrial defect and severe movement disorder in the fly

To elucidate the role of ZnT9 in vivo, we first undertook a functional analysis of the *Drosophila* ZnT9 orthologue (dZnT9). Two independent transgenic lines (1# and 2# lines) of *dZnT9* RNAi and one mutant line were obtained. The expression of *dZnT9* was first determined by qPCR. The transcript of *dZnT9* was significantly decreased in both *dZnT9* RNAi flies (directed by the ubiquitous *Da-GAL4* driver) (Fig. S1A-B). Between the two RNAi lines, RNAi 2# expression suppression was more efficient. The *dZnT9* mutant flies carry an insertion of MiMIC (Minos Mediated Integration Cassette) in the coding exon [27], resulting in virtually non-detectable *ZnT9* expression (Fig. S1C).

**Fig. 2:**
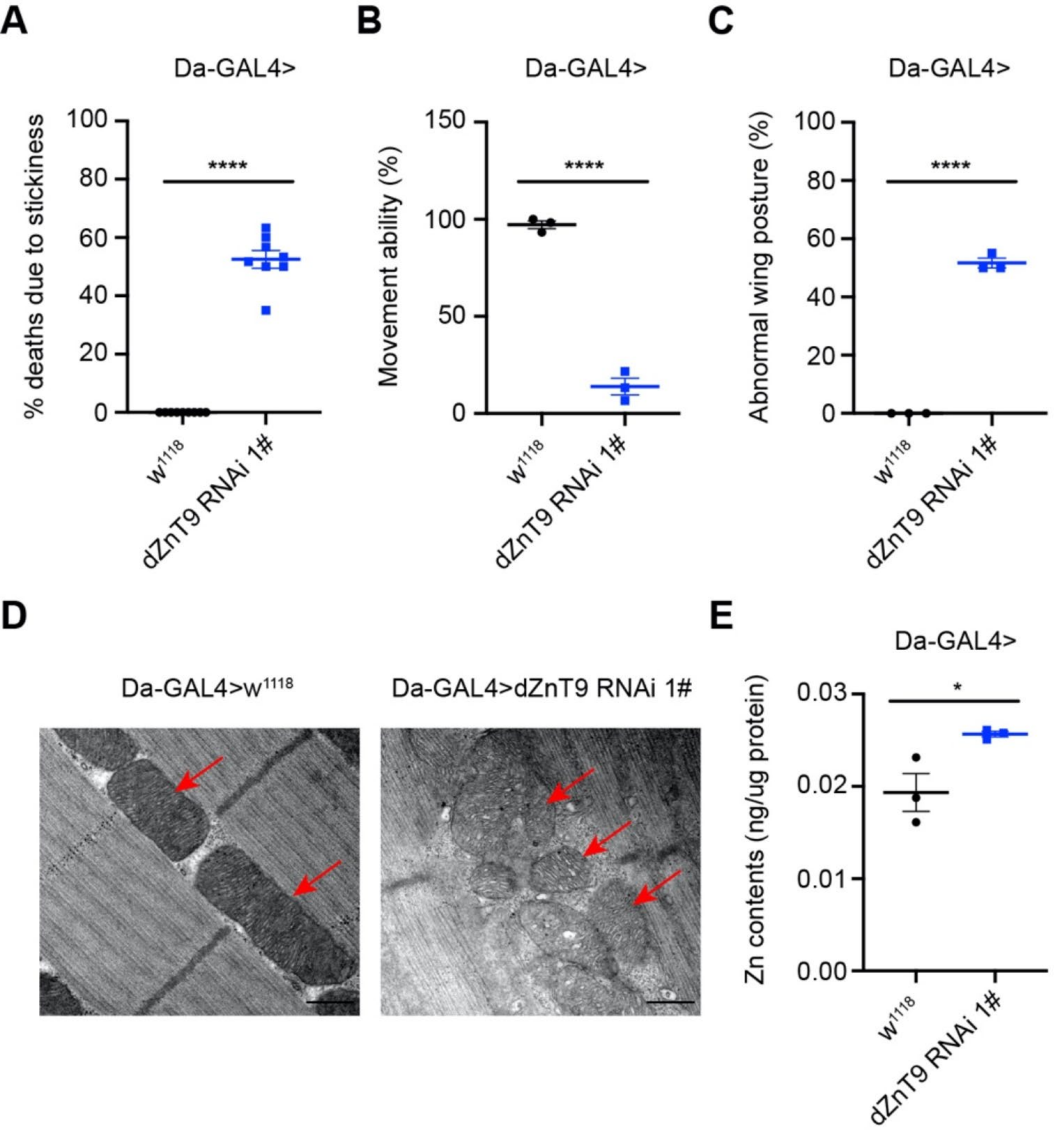
*Drosophila ZnT9* knockdown led to mitochondrial defects, incapacitated movement, and mitochondrial zinc elevation. (**A**) The percentage of flies dying from food stickiness (*n* = 8 per group, each sample includes 60 flies). *dZnT9* knockdown did not result in lethality per se, but some flies died from food-sticking due to motion disability. (**B**) Climbing performances of WT and *dZnT9* RNAi 1# flies (*n* = 3 per group, each sample includes 20 flies). (**C**) The percentage of flies with abnormal wing posture (*n* = 3 per group, each sample includes 20 flies). (**D**) TEM images of mitochondria (marked by red arrows) in the thoracic muscles of WT and *dZnT9* RNAi 1# flies. Scale bar = 500 nm. (**E**) The zinc level of mitochondria from WT and *dZnT9* RNAi 1# flies (*n* = 3 per group)

*dZnT9* knockdown or knockout appeared not to affect much the *Drosophila* at larval stages (Fig. S1D-F). They generally proceeded to pupal formation without much reduction in viability. However, *dZnT9* interruption seriously affected the pupal stage (Fig. S1H-I). Compared to that of *dZnT9* RNAi 2# flies, the eclosion (the process from pupae to adults) rate of *dZnT9* RNAi 1# flies was less dramatically affected (Fig. S1G-H), corresponding to its higher remaining *ZnT9* expression (Fig. S1A-B). In order to analyze the adult phenotype, we first chose the milder *dZnT9* RNAi 1# line because the eclosion rate of *dZnT9* RNAi 1# flies was not as much affected. About 50% of the *dZnT9* RNAi 1# flies got stuck in the food after a few days of rearing (Fig. 2A), implying severe motion disability. Indeed, movement ability measurement confirmed that these alive *dZnT9* RNAi 1# flies displayed apparent movement deficiency (Fig. 2B and Media File S1). Additionally, roughly 50% of them displayed abnormal wing posture, which means they could not close their wings (Fig. 2C). This wing defect could be due to muscle or hinge defects, or even the abnormality of nerve system. The more severe knockdown line, the *dZnT9* RNAi 2# *Drosophila*, died at the pupal stage without eclosed flies (Fig. S1H). *dZnT9*-knockout *Drosophila* resembled largely the more severe form of knockdown: they all died as pupae (Fig. S1I).

During the implementation of the project, two ZnT9 papers came out reporting the functions of the *C. elegans* ZnT9 orthologue (cZnT9). It was shown that the mutant worm was viable with abnormal mitochondrial morphology. Further research demonstrated that cZnT9 influences the mitochondrial zinc level and suggested that cZnT9 effluxes zinc from mitochondria to the cytosol [17–19]. Based on these results, we analyzed the mitochondria in the thoracic muscles of *dZnT9* RNAi 1# flies. The *dZnT9* RNAi mitochondrion was grossly misshapen, and the crista was especially severely disrupted (Fig. 2D). The mitochondrial zinc level in *dZnT9* RNAi 1# flies was then measured by inductively coupled plasma-mass spectrometry (ICP-MS). The zinc level after *dZnT9* knockdown was increased significantly compared with the control (Fig. 2E). Taken together, our results confirm the requirement of ZnT9 in *Drosophila* development and show a unique role for ZnT9 in the homeostatic regulation of mitochondrial zinc.

### Phenotypes of *dZnT9* knockdown can be rescued by mouse ZnT9 or zinc chelation

**Fig. 3:**
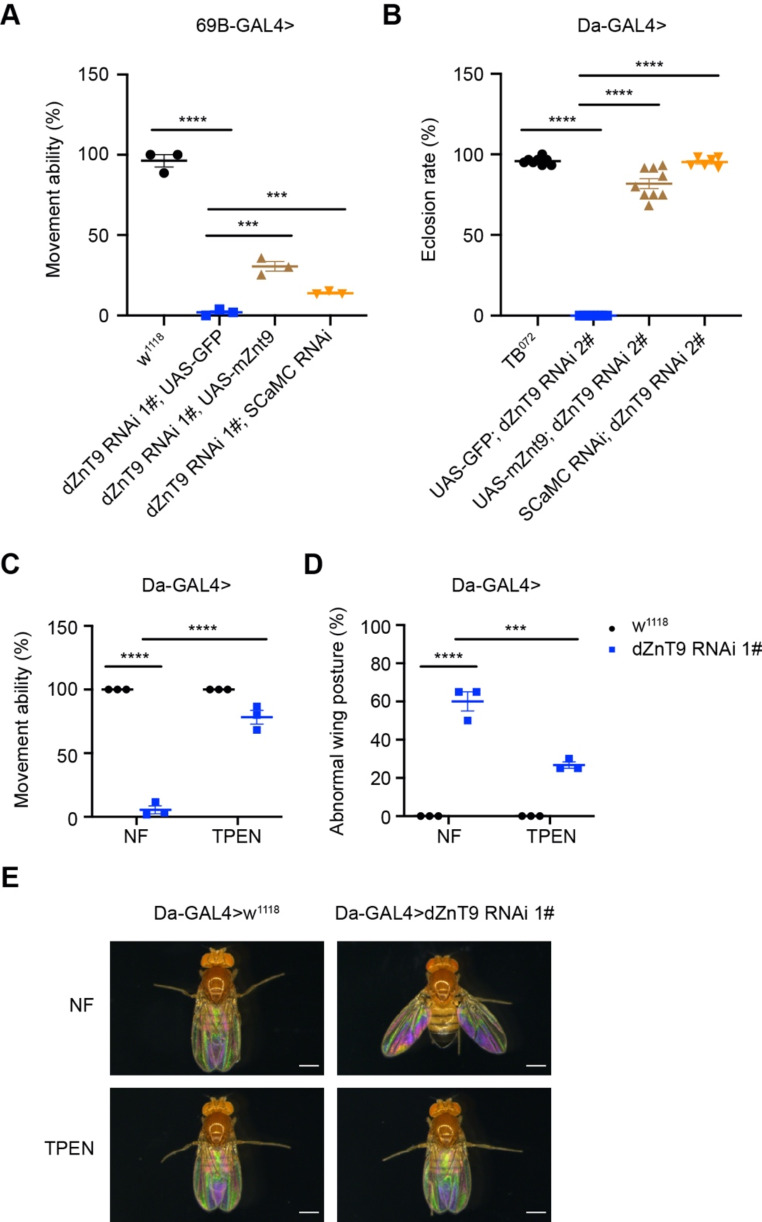
Defects of *Drosophila ZnT9* knockdown could be complemented by mouse ZnT9 or zinc chelator TPEN treatment. (**A**) The movement deficiency of *dZnT9* RNAi 1# flies was rescued by exogenously introduced mZnT9 or endogenous *SCaMC* knockdown. Expression or knockdown was by the UAS-GAL4 system using *69B-GAL4* as the driver (*n* = 3 per group, each sample includes 20 flies). (**B**) The eclosion rate of *dZnT9* RNAi 2# flies was rescued by mZnT9 expression or *SCaMC* RNAi (*n* = 6 per group, each sample includes 60 flies). (**C**) The movement deficiency of *dZnT9* RNAi 1# flies was partially rescued by TPEN (*n* = 3 per group, each sample includes 20 flies). (**D**) The abnormal wing posture of *dZnT9* RNAi 1# flies was partially rescued by TPEN (*n* = 3 per group, each sample includes 20 flies). (**E**) Representative images of wing posture from WT and *dZnT9* RNAi 1# flies reared on normal food (NF) or treated with TPEN (*n* = 6 per group). Scale bar = 0.5 mm

Since ZnT9 was highly conserved among several species, we wanted to know whether mouse ZnT9 expression could rescue the defects of *dZnT9* knockdown. The movement deficiency of *dZnT9* RNAi 1# flies driven by *69B-GAL4*, which expresses in embryonic epiderma and imaginal discs, could be partially rescued by mZnT9 expression (Fig. 3A). Moreover, the eclosion rate of *dZnT9* RNAi 2# flies could also be rescued by mZnT9 (Fig. 3B). Previous findings indicated that SCaMC-2 (SLC-25A25) is a mitochondrial zinc transporter in *C. elegans*, moving zinc from the cytosol to mitochondria [18]. We asked whether the defects of *dZnT9* loss of function could be rescued by *SCaMC* (the orthologue of *SCaMC-2* in the fly) knockdown. The results indicated that the reduction of *SCaMC* could alleviate the movement and eclosion defects of *dZnT9* knockdown flies (Fig. 3A-B).

Given that zinc was accumulated in the mitochondria of *dZnT9* knockdown flies (Fig. 2E), we wanted to know if zinc chelation could confer a similar rescue. TPEN, a permeable zinc chelator, reversed the movement deficiency and abnormal wing posture in *dZnT9* knockdown flies (Fig. 3C-E). These results reaffirmed that the observed *dZnT9* knockdown phenotypes are real (not off-target) and are a consequence of zinc dyshomeostasis (accumulation).

### ZnT9 is indispensable to the early embryonic development of mice

**Fig. 4:**
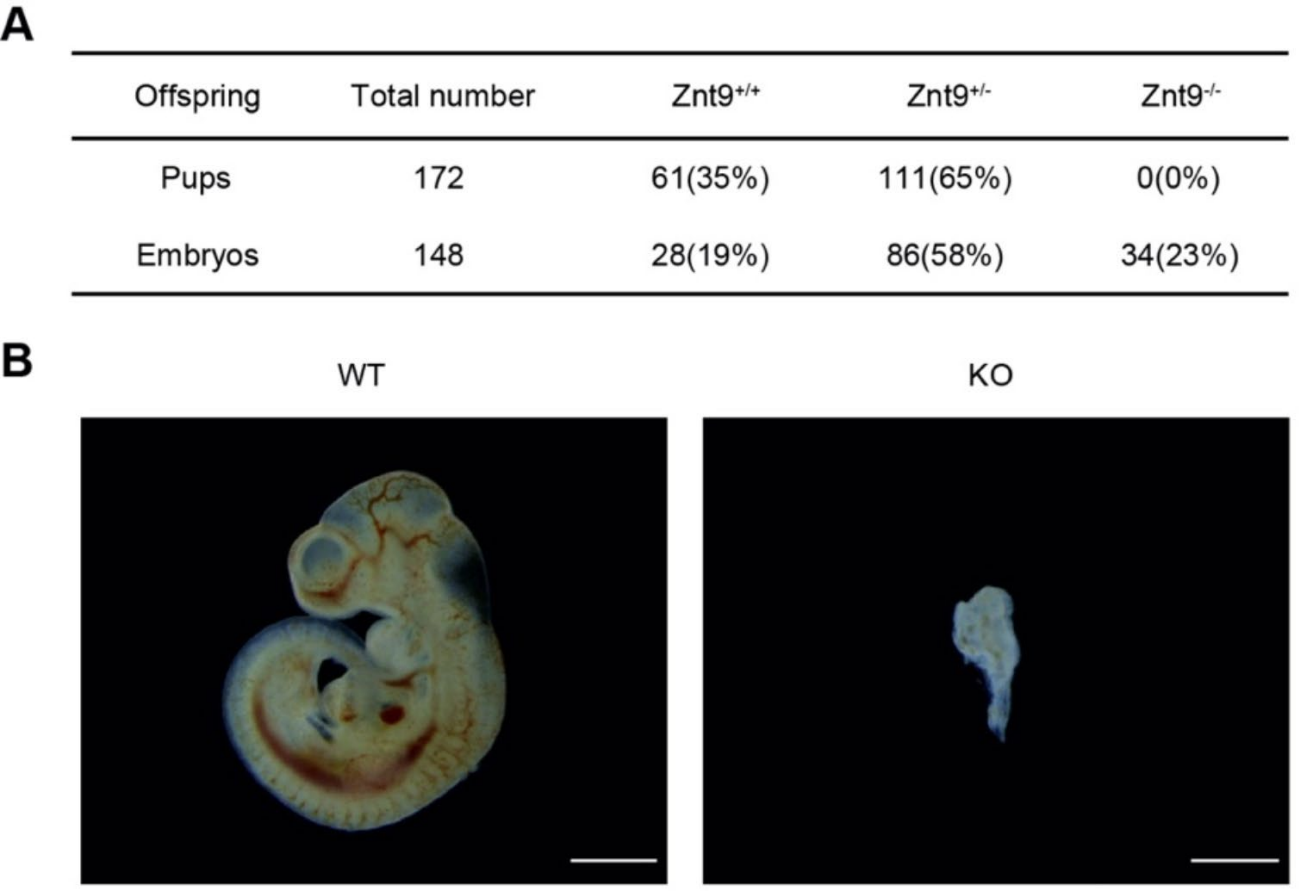
ZnT9 is essential for the early embryonic development of mice. (**A**) Genotype of offspring from heterozygotes matings. (**B**) Morphology of WT and KO embryos at E10.5 dissected free of the yolk sacs (*n* = 9 and 13 for WT and KO, respectively). Scale bar = 1 mm

The ZnT9 function in mammals was addressed by analyses of ZnT9 in the mouse. Global *Znt9* knockout mice were created, but homozygous knockout mice could not be identified in more than 100 pups from heterozygotes matings (Fig. 4A), strongly indicating embryonic lethality. To confirm this assertion and investigate the phenotype in more detail, early embryos were isolated from *Znt9*^*+/−*^ crosses, and we found that the homozygous knockout embryos were lethal before E10.5. At E10.5, the *Znt9*^−/−^ embryos were severely reduced in size and deformed in shape (Fig. 4B), indicating that ZnT9 is essential to the early embryonic development of mice.

**Fig. 5:**
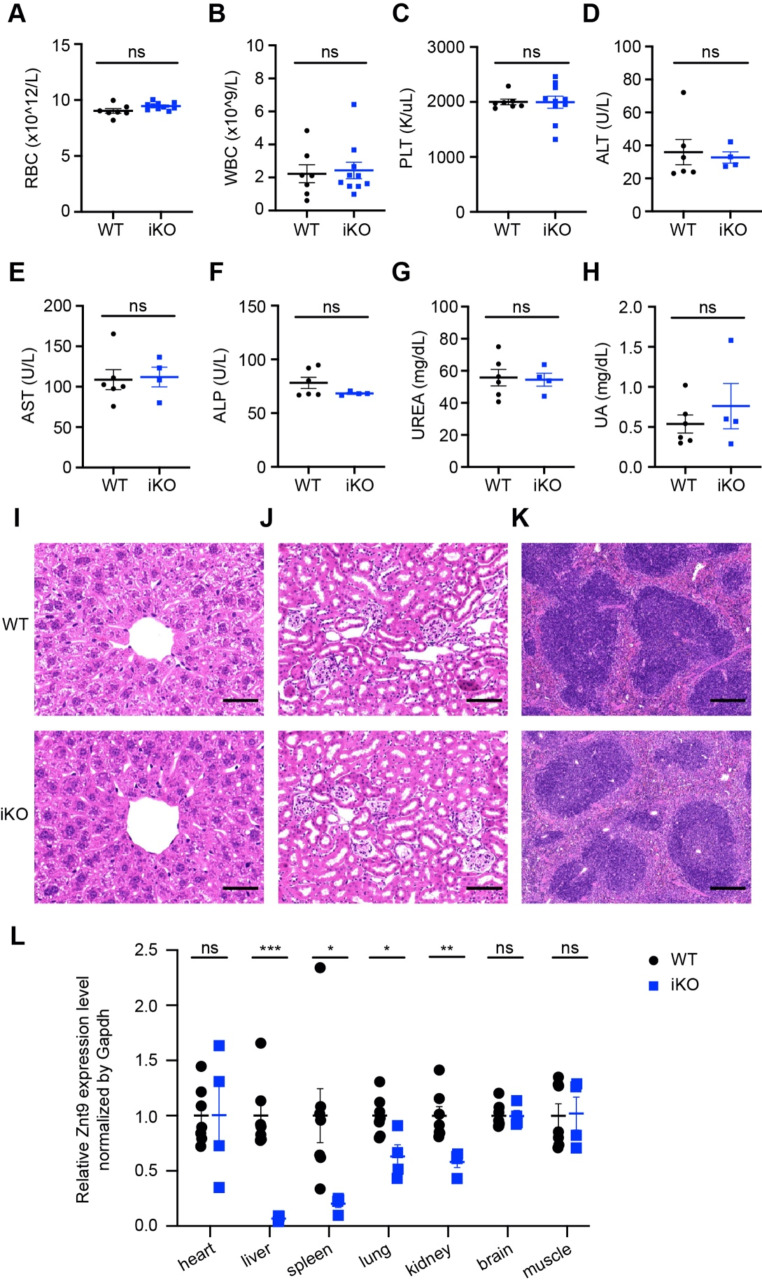
Inducible *Znt9* knockout mice present few overt abnormalities. (**A-C**) Peripheral blood counts of WT and iKO mice (*n* = 7 and 10 for WT and iKO, respectively). (**D-H**) Determination of plasma related indexes of WT and iKO mice (*n* = 6 and 4 for WT and iKO, respectively). (**I**) Representative histological sections of liver from WT and iKO mice (*n* = 5 mice per group). Scale bar = 0.05 mm. (**J**) Representative histological sections of kidney from WT and iKO mice (*n* = 5 mice per group). Scale bar = 0.1 mm. (**K**) Representative histological sections of spleen from WT and iKO mice (*n* = 3 mice per group). Scale bar = 0.2 mm. (**L**) *Znt9* expression levels in different organs of WT and iKO mice (*n* = 7 and 4 for WT and iKO, respectively)

The early lethality phenotype of *Znt9* knockout embryos prompted us to investigate whether there is important function for ZnT9 in the adult. We decided to generate an inducible *Znt9* knockout mice (referred to as *Znt9* iKO mice) using a line of mice ubiquitously expressing the tamoxifen-inducible Cre recombinase under the Rosa26 promoter [28]. To inactivate *Znt9* in adult mice, *Znt9* iKO mice were treated with tamoxifen intraperitoneally at 6 to 8 weeks of age and euthanized 2 months after the treatment. Few abnormalities were noticed. *Znt9* iKO mice were viable and healthy by this time. The peripheral blood counts and plasma-related indexes of *Znt9* iKO mice were also examined. Again, the *Znt9* iKO mice did not present much aberration (Fig. 5A-H). We further performed histological analysis and found no significant abnormalities in the liver, kidney and spleen from *Znt9* iKO mice (Fig. 5I-K). To examine the expression of *Znt9*, i.e., the inducible knockout efficiency, we performed qPCR analysis using different tissues of *Znt9* iKO mice. *Znt9* expression levels in the liver, and spleen were drastically diminished, while in the lung and kidney tissues *Znt9* expression was reduced but not as much affected. *Znt9* expressions in the heart, brain, and muscle were insignificantly suppressed (Fig. 5L). Because the heart, brain, and muscle are primarily mitochondria-enriched tissues, inefficient gene removal in these tissues possibly underlies the lack of phenotypes in the *Znt9* iKO mice. Thus, our inducible KO approach proved not an effective means to analyze ZnT9 function post-early embryonic development. Nevertheless, it shows that the liver and spleen, or more broadly, those tissues not mitochondrion-rich, are not vulnerable to *Znt9* loss.

### Targeted mutagenesis of *Znt9* in the brain leads to dwarfism and lethality

The low efficiency of inducible loss of ZnT9 in mitochondrion-rich organs led us to try tissue-specific *Znt9* knockout. Clinical features of Birk-Landau-Perez syndrome, caused by ZnT9 mutations, are movement disorder, intellectual disability, oculomotor apraxia, developmental regression, and renal insufficiency [20–23]. These characteristics suggested to us that ZnT9 might play a critical role in the nervous system, a mitochondrion-rich tissue. To delineate the role of ZnT9 in the mice brain, we used *Nestin-Cre* to excise the floxed-*Znt9* allele in the whole brain of mice (referred to as *Znt9* cKO mice). To validate the deletion efficiency of *Znt9* cKO, RT-PCR and qPCR were performed, and a significant decrease of *Znt9* expression levels was indeed observed in the *Znt9* cKO brain (Fig. S2A). As an additional control, we also analyzed *Znt9* expression in the liver and kidney tissues and found no significant changes in relative *Znt9* RNA levels of *Znt9* cKO mice (Fig. S2B-C).

**Fig. 6:**
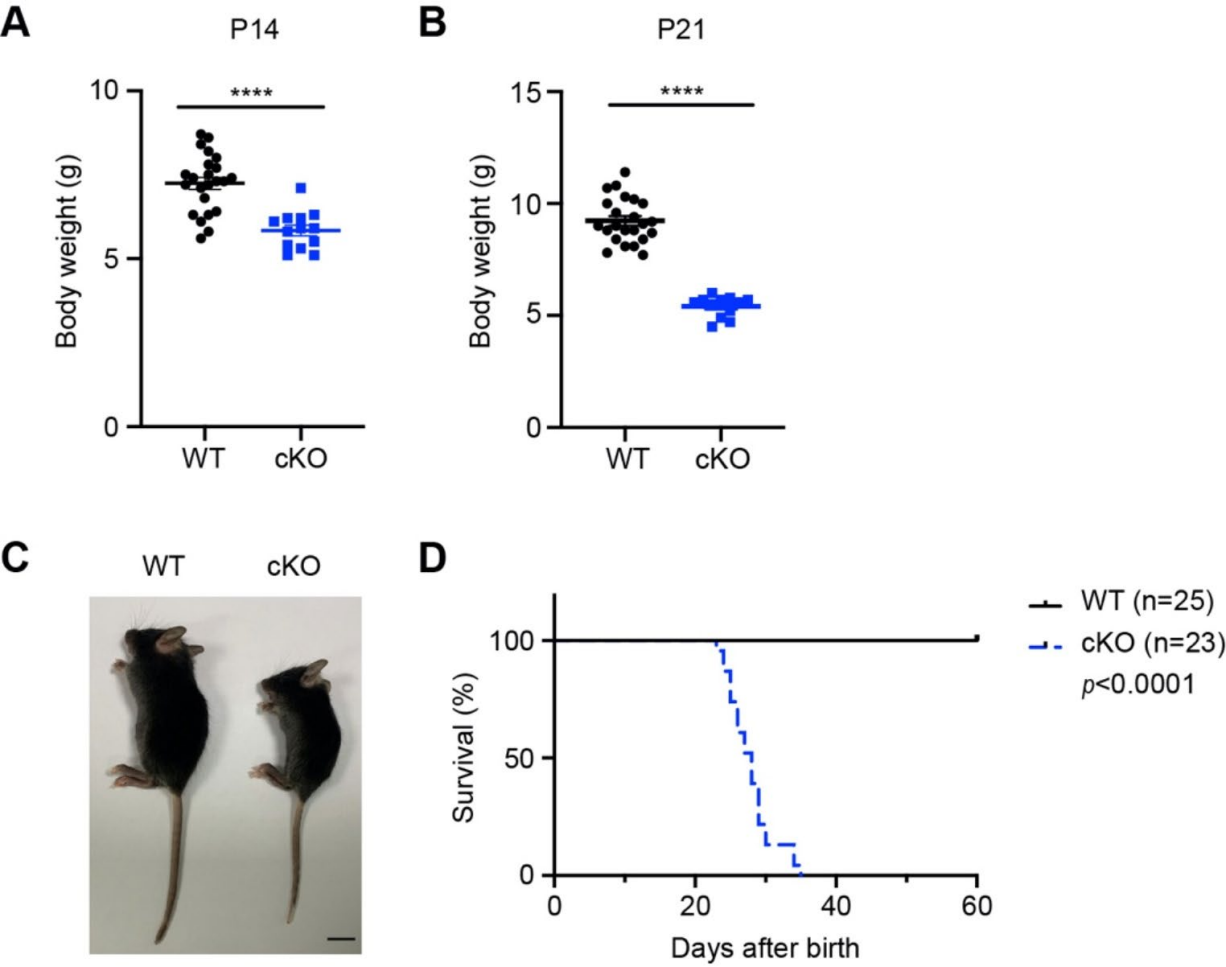
Targeted mutagenesis of *Znt9* in the brain produced severely dwarf and shortly lived mice. (**A**) Body weight of WT and cKO mice at P14 (*n* = 23 and 13 for WT and cKO, respectively). (**B**) Body weight of WT and cKO mice at P21 (*n* = 20 and 18 for WT and cKO, respectively). (**C**) Representative images of WT and cKO mice at P21. Scale bar = 1 cm. (**D**) Kaplan–Meier survival curve of WT and cKO mice (*n* = 25 and 23 for WT and cKO, respectively)

Brain *Znt9* cKO mice could be born without apparent external abnormalities. However, by 2 and 3 weeks of age, *Znt9* cKO mice were much reduced in size and weight (Fig. 6A-C). *Znt9* cKO mice also presented serious movement disorder and tremors, dying at 3 to 5 weeks after birth (Fig. 6D and Media File S2). These results indicate that *Znt9* ablation in the mouse brain results in dwarfism, severe loss of motion ability and a much-shortened lifespan. Interestingly, in the medical field, it is known that severe mitochondrial deficiency sometimes correlates with stunted growth [29]. The exact mechanism still needs to be clarified.

### The GH/IGF-1 axis is severely impaired in the *Znt9* cKO mice

Since no reduction in growth was observed in worms and flies, we subsequently asked how ZnT9 affected the size of mammals. Is it due to a growth hormone (GH) shortage? Pituitary glands, a part of the brain, are composed of neurohypophysis or the posterior lobes, and adenohypophysis, including the anterior and intermediate lobes [30]. Each cell type of adenohypophysis is characterized by the hormone it secretes. There are seven different hormones in the adenohypophysis: growth hormone produced by somatotropes, prolactin (PRL) produced by lactotropes, thyroid-stimulating hormone (TSH) produced by thyrotropes, adrenocorticotropic hormone (ACTH) produced by corticotropes, luteinizing hormone (LH) and follicle-stimulating hormone (FSH) produced by gonadotropes, and melanocyte-stimulating hormone (MSH) produced by intermediate lobe melanotropes [31]. GH is a key regulator hormone that plays an important role in body growth [32]. The production of IGF-1 by hepatocytes is regulated by GH [33]. Previous studies have established that the GH/IGF-1 axis plays a major role in controlling postnatal growth [32].

**Fig. 7:**
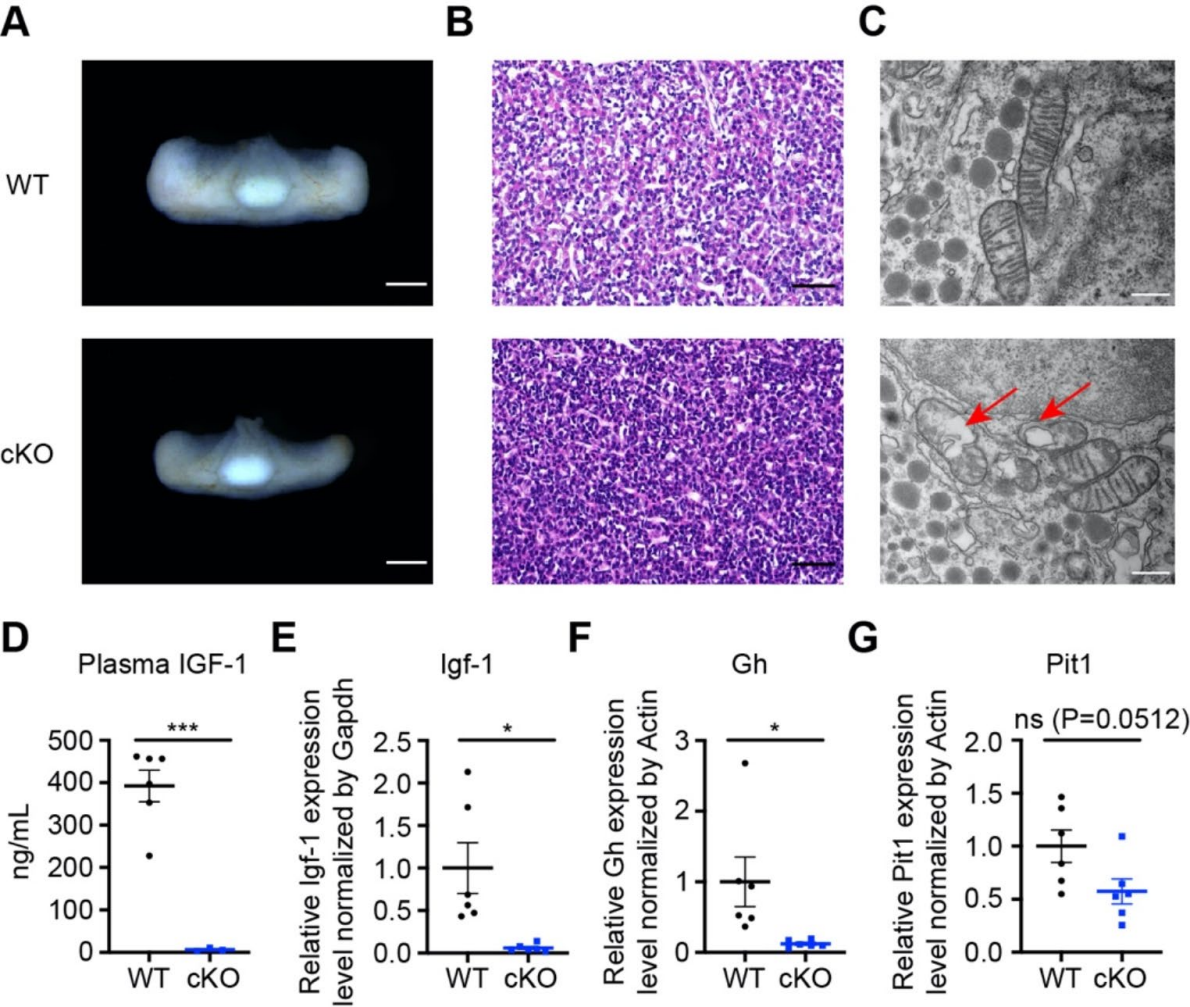
Targeted mutagenesis of *Znt9* in the brain gravely impaired the GH/IGF-1 axis. (**A**) Representative images of pituitaries from WT and cKO mice at P21 (*n* = 10 and 7 for WT and cKO, respectively). Scale bar = 0.5 mm. (**B**) Representative histological sections of pituitaries from WT and cKO mice (*n* = 3 mice per group). Scale bar = 0.05 mm. (**C**) TEM images of mitochondria from WT and cKO pituitaries (*n* = 3 mice per group). Red arrows mark defective mitochondria from cKO mice. Scale bar = 200 nm. (**D**) Elisa analysis of plasma IGF-1 levels (*n* = 6 and 3 for WT and cKO, respectively). (**E**) qPCR for *Igf-1* in the liver of WT and cKO mice (*n* = 6 mice per group). (**F-G**) qPCR for *Gh* (F) and *Pit1* (**G**) in the pituitaries of WT and cKO mice (*n* = 6 per group). A sample was pooled from three pituitaries of the same genotype

To examine possible defects in the pituitary glands, we dissected the tissue. We found that the pituitary glands of *Znt9* cKO mice were smaller compared to wildtype (Fig. 7A). However, the reduction in size was roughly proportionally to the overall body decrease. Again, the posterior and anterior lobes areas of *Znt9* cKO mice were all in proportion smaller than the control (Fig. S3A-C). While the ratio of total area and anterior lobes area to body weight of *Znt9* cKO mice showed no significant change, the ratio of posterior lobes area to body weight increased (Fig. S3D-F). Histological sections of *Znt9* cKO pituitaries showed no apparent defects (Fig. 7B). However, the mitochondria from the *Znt9* cKO pituitaries exhibited disrupted cristae in morphology (Fig. 7C). Since the GH/IGF-1 axis controlled the body growth, the *Igf-1* and *Gh* expression levels were measured. We found that the expression levels of *Igf-1* and *Gh* both decreased dramatically in *Znt9* cKO mice to almost an undetectable level (Fig. 7D-F). How could GH be so much affected? PIT1 is a major transcription factor and plays an essential role in the differentiation of somatotropes, lactotropes, and thyrotropes [31]. We then measured *Pit1* expression in *Znt9* cKO mice. *Pit1* expression was downregulated slightly but not dramatically (Fig. 7G), at least not affected to the extent as the *Gh*. These results indicate that the GH/IGF-1 axis of *Znt9* cKO mice is severely impaired, possibly partially due to *Pit1* decrease, but other factors may also be involved.

One previous work reported that *Nestin-cre* is not expressed in the pituitary glands [34]. Since the GH/IGF-1 axis was impaired in *Znt9* cKO mice, we wanted to explore if the defect was due to the loss of *Znt9* in the pituitary or the hypothalamus. qPCR and genomic PCR were performed to measure the knockout efficiency in the pituitary. The result indicated that *Znt9* was not eliminated effectively in the pituitary of *Znt9* cKO mice; most animals did not carry significant *Znt9* deletion (Fig. S2D-E). The genomic PCR for hypothalamus from *Znt9*^*fl/fl*^, cKO/+ and cKO mice was also performed. *Znt9* was deleted efficiently in the hypothalamus (Fig. S2F). Therefore, it seems the loss of GH/IGF signaling was not due to the pituitary per se, but possibly a result of the hypothalamus-pituitary signaling.

### *Znt9* cKO is not associated with dopamine deficiency but results in a decrease in multiple neuroactive ligand − receptor signalings

In addition to being small in size, *Znt9* cKO mice also displayed movement disorder and tremors, especially in the four limbs (Media File S2). These behaviors are reminiscent of the features of Parkinson’s disease. The main cause of Parkinson’s disease is impairment of dopaminergic neurons in the substantia nigra, due to the formation of protein inclusions named Lewy bodies (LBs) there, leading to a decrease of the dopamine level in the striatum [35]. We have previously shown that cytosolic zinc levels could affect tyrosine hydroxylase (TH) activity, leading to dopamine level change, through zinc-iron competition for TH [36]. To address whether the motion abnormality and tremors in *Znt9* cKO mice arise from dopamine deficiency, we measured the dopamine level in the striatum of *Znt9* cKO mice. No significant change was found (Fig. S4A). The expression levels of tyrosine hydroxylase in the substantia nigra of *Znt9* cKO mice also presented no significant difference (Fig. S4B-C). Consistently, Nissl staining of *Znt9* cKO striatum did not show obvious defects (Fig. S4D). These data suggest that the tremors of *Znt9* cKO mice are not due to a decrease of dopamine in the striatum.

The observation that *Znt9* mutation translates to GH signaling loss but the upstream *Pit1* is not as affected indicates that other upstream components may be involved. RNA-seq data analysis of the *Znt9* cKO mice brain revealed a striking feature: the downregulation of neuroactive ligand − receptor interaction stands out (Fig. S5). In addition, abundant signaling pathways changed in *Znt9* cKO mice brain (Fig. S6). This suggests that *Znt9*-loss-resulted mitochondrial dysfunction is associated with multiple neuro-signaling defects.

### Mitochondrial ETC activity is impaired by zinc accumulation

**Fig. 8:**
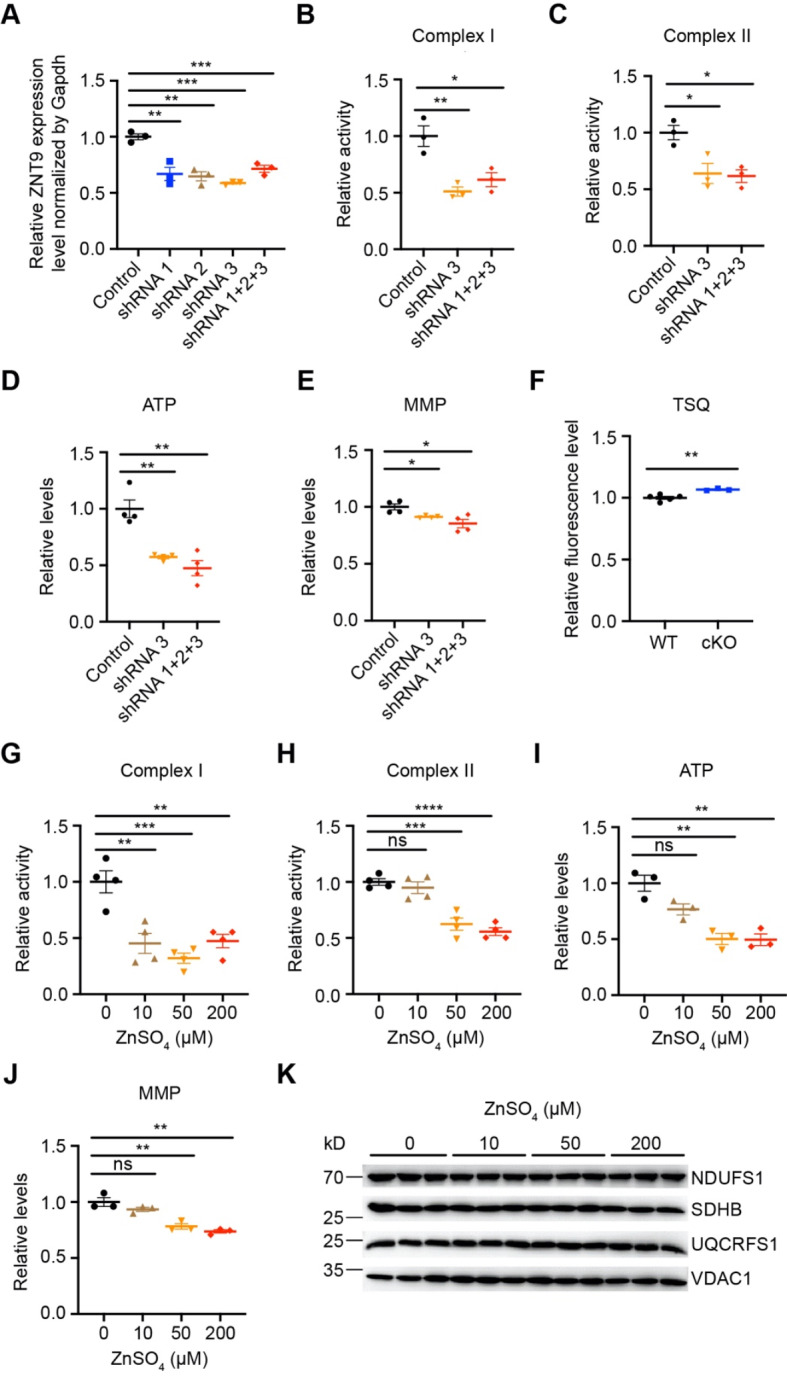
Mitochondrial ETC activity is vulnerable to zinc accumulation. (**A**) Transcripts for *ZnT9* from 293T cells were quantified by qPCR 72 h after transfection as indicated. (**B**) The relative activity of complex I in 293T cells was examined 72 h after transfection as indicated. (**C**) The relative activity of complex II in 293T cells was examined 72 h after transfection as indicated. (**D**) The relative ATP levels in 293T cells were measured 72 h after transfection as indicated. (**E**) The relative mitochondrial membrane potential levels in 293T cells were measured 72 h after transfection as indicated. (**F**) The mitochondria from WT and *Znt9* cKO mice brain were isolated and the labile zinc content was determined using TSQ. (**G**) The relative activity of complex I after the mitochondria isolated from 293T cells were incubated with different concentrations of ZnSO_4_ for 2 h. (**H**) The relative activity of complex II after the mitochondria purified from 293T cells were incubated with different concentrations of ZnSO_4_ for 2 h. (**I**) 293T cells were incubated with different concentrations of ZnSO_4_ for 24 h, and the relative ATP levels were examined using the isolated mitochondria. (**J**) 293T cells were incubated with different concentrations of ZnSO_4_ for 24 h, and then the relative mitochondrial membrane potential levels were determined. (**K**) Expression of NDUFS1, SDHB, and UQCRFS1 was examined by western blotting after the mitochondria were incubated with different concentrations of ZnSO_4_ for 2 h

How does ZnT9 loss affect mitochondrial functions? Does zinc accumulation inhibit the respiratory complex (the electron transport or the ETC) activity? To answer these questions, we first knocked down *ZnT9* in 293T cells by transfecting three separate shRNAs. *ZnT9* expression analyses by qPCR revealed that *ZnT9* was downregulated significantly in 293T cells after shRNA transfection either separately or together (Fig. 8A). The mitochondrial complex activity was then determined by transfecting the more effective shRNA 3# or the three shRNAs together. Complex I and II activities decreased dramatically after *ZnT9* knockdown in 293T cells (Fig. 8B-C). Furthermore, ATP contents and mitochondrial membrane potential (MMP) were also measured. Loss of *ZnT9* led to lower ATP contents and mitochondrial membrane potential (Fig. 8D-E).

To confirm that the mitochondrial labile zinc in *Znt9* cKO mice brain is elevated, we isolated mitochondria and measured the free zinc level with TSQ, a cell-permeable zinc probe. Loss of *Znt9* led to mitochondrial zinc increase (Fig. 8F). This is consistent with previous publications showing increased mitochondrial zinc levels [17–19]. The impact of zinc on mitochondrial ETC activity could be direct or indirect. For example, zinc accumulation could affect iron-sulfur (Fe-S) formation or ETC complex assembly. We therefore analyzed the consequence of mitochondria, isolated from 293T cells, after a short-time zinc treatment. Short-time treatment of mitochondria with zinc similarly led to suppression of complex I and II activities (Fig. 8G-H). 293T cells were treated with zinc for 24 h and then the mitochondria were isolated. Zinc supplementation resulted in decreased ATP contents and mitochondrial membrane potential (Fig. 8I-J). However, the complex expression levels and Fe-S synthesis appeared not significantly changed (Fig. 8K). These data indicate that zinc could exert an immediate direct inhibition on ETC activity.

## Discussion

Our studies revealed that ZnT9 plays a critical role in the development of flies and mice embryos. Lack of *ZnT9* led to mitochondrial zinc dyshomeostasis and movement defect in both the flies and mice. ETC activities were obviously suppressed, and mitochondrial morphology was gravely disturbed after *ZnT9* loss. Deletion of *Znt9* in the brain resulted in dwarfism and reduced lifespan in mice, indicating its crucial role in orchestrating a network of players involved in GH regulation. Our work provided clues for further understanding of ZnT9 in mitochondrial zinc homeostasis, its in vivo role in insects and mammals, and helps understand the etiology associated with the ZnT9-associated cerebro-renal syndrome.

It is interesting to see that during evolution, blocks of ZnT9 across most of the protein are conserved. This is unusual for ZIPs/ZnTs, where the most conserved regions are usually the critical transmembrane domains. The conserveness of these regions out of TMs, most of which located at the large C- termini, suggests that ZnT9 is associated with other important functions besides simply zinc transport. It would be interesting to decipher these in details in future studies.

Given the critical function of ZnT9, evidenced by the early embryonic lethal phenotype of *Znt9* knockout mice, how do we reconcile that with the observation of the severe but viable phenotype in human patients? The first *ZnT9* mutation was reported in a consanguineous Bedouin kindred [20]. The same mutation was recently identified in another two patients [21]. This change (c.1049delCAG, pAla350del; red arrow in Fig. 1A) would delete an amino acid in the putative TM IV. The deleted amino acid is partially conserved during evolution. The remaining TM IV (20 a.a.) still seems large enough to span the whole membrane, so a residual function of ZnT9 is possible. Another *hZnT9* mutation was found in eight individuals from four unrelated families. Specifically, the homozygous variant in *ZnT9* was detected in three individuals from two unrelated British Pakistani families (c.1253G > T, p.Gly418Val, blue arrow in Fig. 1A) [23]. Again, the glycine residue between TM V and TM VI is semi, but not absolutely, conserved. In all likelihood, the two mutations just mentioned would probably only lead to partial loss of function to hZnT9. Among the remaining five patients, four individuals carry the same mutation as the first case reported by Perez et al. The last pathogenic variant (c.1413 A > G, p.Ser471=/c.1083dup, p.Val362Cysfs*5) was found in a European Australian family. In this patient, one allele (p.Val362Cysfs*5) carries a frame-shift mutation in the middle of the protein and should be null, but the other allele is a synonymous mutation hypothesized to impact the splicing [23]. Since alternative splicing forms, including the original normal splicing form, could exist, this patient might also carry some residual normal ZnT9 protein.

However, Kleyner and colleagues described a ZnT9-associated cerebro-renal syndrome in a non-consanguineous family with compound heterozygous frameshift mutations (c.40delA, p.Ser14Alafs*28/c.86_87dupCC, p.Cys30Profs*13). These two alleles both stop shortly after the ATG start, and it is expected to obtain short peptides [22]. Careful examination of the sequence revealed that immediately downstream of the expected translational stop lies another ATG (Fig. 1A). This second ATG could serve as a restart signal for the prematurely stopped translation and result in an in-frame translation leading to an N-terminal truncated product, which would retain all the conserved blocks of residues including the TMs and the large N- and C- loops. In spite of this, a functional region, i.e., the original mitochondrial signal, would be lost. It is not known how much function would be maintained with the N-truncated protein, nor how much of the protein might end up in the mitochondrion after a mitochondrion-targeted translation followed by a restarted translation product.

The same study by Kleyner and colleagues also reported an overall mutation rate of around 0.4% in the general population [22]. Given the conserved nature of the protein, it is estimated up to 1/100,0000 pregnancies might be affected. However, it is possible that detrimental mutations might result in early embryonic disappearance. Hence, lower frequency may be observed in live births.

Remarkably, though both the fly and mice *Znt9* mutants are lethal, and in the case of mice, *Znt9* loss leads to the more extreme phenotype-early disappearance of the embryos, the worm *ZnT9* mutant was reported OK. It is possible that the worm is not particularly vulnerable to mitochondrial disruption. Consistently, in mice, loss of *Znt9* in the liver and spleen, as happened in *Znt9* iKO mice, presents no apparent abnormality. So, even in mammals, loss of ZnT9 may only show overt phenotypes in a set of tissues that demand robust mitochondrial functions.

ZnT proteins are generally considered to transport zinc from the cytosol to intracellular compartments or the extracellular space, reducing zinc in the cytosol [37]. In this respect, the mitochondrion-resident ZnT9 moves zinc in the opposite orientation: from the mitochondrion to the cytosol. However, from the perspective of the mitochondrion, a remnant of ancient symbiont cells, ZnT9 still acts to efflux zinc out of the matrix, which is analogous to the cytosol. Mechanistically, ZnT transporters may be proton-driven, and the proton gradient in mitochondria is opposite to that of other organelles, such as the Golgi apparatus [19]. The direction of the proton gradient in mitochondria may enable the opposite orientation of zinc movement driven by ZnT9 [19].

Notably, the initially thought zinc transporter ZnT10 turns out to be a manganese transporter, and mutations in human ZnT10 cause a Parkinson-like hypermanganesemia [38–40]. Therefore, the substrates of ZnTs may not be limited to zinc only. Since ZnT9 loss causes mitochondrial zinc accumulation, and in addition, dZnT9 loss can be rescued by the addition of TPEN, a zinc chelator, or mammalian ZnT9 expression, we conclude that the defect of dZnT9 loss indeed arises from zinc dyshomeostasis and ZnT9 functions are conserved during evolution. Along with this thinking, ZnT9 is an essential gatekeeper of mitochondrial zinc homeostasis, which affects ETC activity. It is worth pointing out that zinc’s effect on ETC has been reported before, especially on the bc_1_ complex [16]. We showed here that complex I and II activity could also be affected. It seems, therefore, that zinc’s effect on the ETC is likely quite broad.

## Materials and methods

### Fly stocks

Flies were maintained on standard cornmeal media or treated with zinc chelator (N, N,N′,N′-tetrakis (2-pyridylmethyl) ethylenediamine, TPEN). The concentration of TPEN (TCI (Shanghai) Development Co., Ltd., Shanghai, China) was 50 µM.

Fly strains used in this study are listed as follows: *Da-GAL4* (Bloomington #8641), *69B-GAL4* (Bloomington #1774) and *dZnT9* mutant (Bloomington #56313) were obtained from the Bloomington Drosophila Stock Center. *dZnT9* RNAi 1# (VDRC #4654), *SCaMC* RNAi (VDRC #29439) and *SCaMC* RNAi (VDRC #108078) were obtained from the Vienna Drosophila RNAi Center. *dZnT9* RNAi 2# (THFC #2312) was purchased from Tsinghua Fly Center. *UAS-mZnt9* transgenic flies were generated in this study. *mZnt9* was PCR-amplified from mouse cDNA and then cloned into the vector pUAST-attB using the following primers: F: 5′-CGGAATTCGCCACCATGTTTCCGGGCTTGGCC-3′, R: 5′-CCCTCGAGCTATAGGATCTCAAGGTCTAC-3′. Transgenic flies were made by microinjection of the construct into embryos with a phiC31 transgene.

### Fly eclosion assays

Female virgins of *Da-GAL4* were crossed with transgenic flies and the progeny were reared on standard cornmeal media. There were 60 larvae each vial. The number of pupae and emerged adults of each vial was counted for percentage calculation and labelled as pupation rate and eclosion rate respectively. Note that there were transgenic flies dying from weak movement ability and they were adhered to the media which was sticky. The number of these adhered flies was also counted for percentage calculation and labelled as % deaths due to stickiness.

### Mitochondria isolation

The mitochondria were isolated as described previously [41]. Third instar larvae were homogenized in mitochondria isolation buffer containing 250 mM sucrose, 5 mM ethylene glycol-bis(2-aminoethylether)-N, N,N′,N′-tetraacetic acid (EGTA), and 10 mM Tris–HCl. The supernatant was collected after centrifuging at 1000 g for 10 min twice and centrifuged twice at 10,000 g for 15 min to obtain pellet. The pellet was resuspended in isolation buffer and protein concentration was determined by the BCA Protein Assay Kit (Thermo Scientific).

### Metal content assay

To measure the zinc content of the flies, third instar larvae were collected and the mitochondria were isolated. The samples were incubated with HNO_3_ and then digested by microwave. The metal content was determined by inductively coupled plasma-mass spectrometry (ICP-MS) at Research Center for Eco-Environmental Sciences, Chinese Academy of Sciences. All data were normalized by protein levels.

### Behavioral assessment

The locomotion assay was performed as described previously with minor modification [42]. The emerged adults were collected and 20 flies were placed in a vial. The number of flies with abnormal wing posture was counted for percentage calculation. The abnormal wing posture was imaged by a stereoscope (MZ10F, Leica, Germany). For climbing ability analysis, the vial was gently tapped and the number of flies climbing over 7 cm in 7 s was measured. Each vial was tested for 3 times. Climbing activity was determined by normalizing the climbing counts in each group against the mean number. The percentage represents climbing activity. Males were used in this test.

### RNA isolation and quantitative real-time PCR

Total RNA was isolated from third instar larvae using TRIzol reagent (Invitrogen, Carlsbad, CA, USA). cDNA was reverse-transcribed from 1 µg total RNA with TransScript One-Step gDNA Removal and cDNA Synthesis SuperMix (TransGen Biotech Co., Beijing, China) according to the manufacturer’s instructions. Quantitative real-time PCR was performed on LightCycler480II (Roche, Switzerland) using PerfectStart Green qPCR SuperMix (TransGen Biotech Co., Beijing, China). Fold change was determined by comparing target gene expression with the reference gene expression (*rp49*) under the same conditions. The primers used for qPCR were: *dZnT9*, GAGCTGACCCGTTCGTATCTG and CGATCAATTTCGCCGCCCA; *rp49*, GCACCAAGCACTTCATCCGC and CCGTAACCGATGTTGGGCATC.

### Bioinformation analysis

The amino acid sequences of ZnT9 from *C. elegans*, *D. melanogaster*, *D. rerio*, *M. musculus* and *H. sapiens* were obtained from National Center for Biotechnology Information (NCBI) (https://www.ncbi.nlm.nih.gov) and were aligned using Jalview version 2.11.0 [43–45]. The mitochondrial presequence was predicted using MitoFates [46]. The transmembrane domains were predicted using DeepTMHMM [47].

### Transmission electron microscopy

The thoraces of male adult flies were dissected and fixed using 2% paraformaldehyde and 2.5% glutaraldehyde. The samples were washed with 0.1 M PB at room temperature for 3 times, 15 min each. The tissue blocks were fixed using a specially made osmic acid (1% osmic acid + 1.5% potassium hexacyanoferrate) for 1 h and stained with 1% uranyl acetate at room temperature for 1 h. Then the samples were dehydrated and infiltrated with resin and propylene oxide, following by embedding at 60 °C. The resin blocks were cut into 70 nm sections with a microtome (EM UC6, Leica, Germany) and the sections were observed with a H-7650B TEM (HITACHI, Japan) operating at 80 kV.

### Mouse strains

Global *Znt9* knockout mice (C57BL/6J) were created using the CRISPR/Cas9 system by Cyagen Biosciences Inc (Guangzhou, China). Guide RNAs targeting *Znt9* exons 3 and 9 were designed. *Znt9*^*fl/fl*^ mice (C57BL/6J) were generated using the CRISPR/Cas9 system by Cyagen Biosciences Inc (Guangzhou, China). *Znt9* exons 4 and 7 were flanked by loxP sites.

To generate an inducible *Znt9* knockout mice model, *Znt9*^*fl/fl*^ mice were crossed with *Rosa-Cre* transgenic mice which express a tamoxifen inducible Cre recombinase under the Rosa26 promoter [28]. The resulting *Znt9*^*fl/+*^;*RosaCre*^*+/−*^ mice were then crossed with *Znt9*^*fl/fl*^ mice to obtain *Znt9*^*fl/fl*^;*RosaCre*^*+/−*^ mice. *Znt9*^*fl/fl*^;*RosaCre*^*+/−*^ mice were crossed with *Znt9*^*fl/fl*^ mice to obtain *Znt9*^*fl/fl*^;*RosaCre*^*+/−*^ study subjects and their control littermates (*Znt9*^*fl/fl*^ mice). To delete the floxed-*Znt9* gene, tamoxifen (75 mg/kg body weight, ip) was injected 5 consecutive days into male mice aged 6 to 8 weeks. Mice were euthanized 2 months after tamoxifen treatment.

To delete *Znt9* in the brain, *Znt9*^*fl/fl*^ mice were crossed with *Nestin-Cre* transgenic mice (gift from Prof. Ligong Chen, Tsinghua University). The resulting *Znt9*^*fl/+*^;*NestinCre*^*+/−*^ mice were then crossed with *Znt9*^*fl/fl*^ mice to obtain *Znt9*^*fl/fl*^;*NestinCre*^*+/−*^ study subjects and their control littermates (*Znt9*^*fl/fl*^ mice, *Znt9*^*fl/+*^ mice and *Znt9*^*fl/+*^;*NestinCre*^*+/−*^ mice). *Znt9*^*fl/+*^;*NestinCre*^*+/−*^ mice were referred to as cKO/+ mice. The mice used in the experiments were 19–21 days old. Females and males were not distinguished and both used in the experiments.

No more than 6 mice were housed in one cage. All animals were maintained on a 12/12-h light/dark cycle at 20–26 °C with around 40–70% humidity, and give ad libitum access to standard food and water. All mice were housed in the same room and the same cage rack in similar living conditions. When collecting tissues in laboratory, control and experimental animal groups were sacrificed in a random order. Mice were assigned to each group according to their genotype. The sample size was decided on the basis of obtaining reliable and meaningful information, and at the same time, keeping the number of used animals to a minimum. Investigators were not blinded to group assignments since the comparisons were objective and quantitative except for the assessment of histological sections. All experiments were approved by the Institutional Animal Care and Use Committee (IACUC) of Tsinghua University (Beijing, China).

### Embryos isolation

Study subjects *Znt9* homozygous knockout mice (*Znt9*^*−/−*^ mice) were obtained from intercrossed *Znt9* heterozygous knockout mice (*Znt9*^*+/−*^ mice) and were compared with their control littermates (*Znt9*^*+/+*^ mice). Embryonic staging was determined by standard methods counting the morning on which the vaginal plug was found as E0.5. E10.5 embryos were dissected and imaged by a stereoscope (MZ10F, Leica, Germany).

### Western blot analysis

Substantia nigra were collected and homogenized at 4 °C in RIPA Lysis Buffer (Servicebio, Wuhan, China) containing 1% protease inhibitor cocktail (Bimake, Shanghai, China). After centrifugation, the supernatants were separated on 10% SDS-PAGE electrophoresis and transferred onto PVDF membranes (Millipore, Watford, UK).

The primary antibody used in this study was rabbit anti-TH, 1:1000 (Merck, Darmstadt, Germany). The control mouse anti-Actin was obtained from Proteintech (Rosemont, USA). Secondary antibodies were HRP-conjugated goat anti-mouse IgG and goat anti-rabbit IgG (EASYBIO, Beijing, China).

### Histological analysis

The tissues were collected and fixed using 4% paraformaldehyde. The tissue sections were prepared using Pathology slicer (RM2016, Shanghai Leica Instrument Co., Ltd). The paraffin sections were dehydrated and stained with Hematoxylin and Eosin or toluidine blue. The sections were scanned using 3DHISTECH Digital Pathology System (Pannoramic SCAN, 3DHISTECH, Hungary). The histological slides were assessed by veteran researchers who were blinded to the group allocations.

### Blood analysis

Peripheral blood samples were collected using EDTA-coated microtubes and blood counts were determined with an automated blood analyzer (XN-1000 V, Sysmex, Japan).

Peripheral blood samples were collected using heparin-coated tubes and centrifuged at 2500 rpm for 15 min to obtain plasma. Plasma related indexes were determined using Automatic Biochemistry Analyzer (Cobas 6000 c 501, Roche, Switzerland).

To measure the IGF-1 levels in plasma, the Mouse/Rat IGF-I/IGF-1 Quantikine ELISA Kit (R&D Systems, Inc., Minneapolis, USA) was used according to the manufacturer’s instructions.

### HPLC analysis of brain dopamine content

Striatum was dissected and weighed. The samples were homogenized in 80% (v/v) HPLC-grade methanol (pre-chilled at -80 °C) on dry ice, followed by vortexing for 1 min at 4–8 °C and then incubation at -80 °C overnight. After thawing, the samples were centrifuged at 14,000 g for 20 min using a refrigerated centrifuge at 4 °C and the same volume of supernatant was transferred to a fresh tube. The samples were lyophilized using CentriVap Concentrators (Labconco, USA). Dopamine contents were determined using ACQUITY UPLC H-Class system which was coupled a 6500plus QTrap mass spectrometer (AB SCIEX, USA) and equipped with a heated electrospray ionization (HESI) probe. Dopamine contents presented were normalized by sample weights.

### Statistics

All data in this study are presented as the means ± SEM and no data were excluded from analysis. The data were analyzed using GraphPad Prism (version 8.4.0; La Jolla, CA, USA). Data were analyzed by unpaired t-test between two groups and ANOVA was used to compare three or more groups. P values less than 0.05 were considered statistically significant (∗*P* < 0.05, ∗∗*P* < 0.01, ∗∗∗*P* < 0.001, and ∗∗∗∗*P* < 0.0001).

### Electronic supplementary material

Below is the link to the electronic supplementary material.

Supplementary Material 1

Supplementary Material 2

## Data Availability

The datasets used and/or analyzed during this study are available from the corresponding author on reasonable request.
