# Supplementary material for: SLC30A9: an evolutionarily conserved mitochondrial zinc transporter essential for mammalian early embryonic development
Source: Cell Mol Life Sci. 2024 Aug 19;81(1):357. doi: 10.1007/s00018-024-05377-y (PMC11335279; doi:10.1007/s00018-024-05377-y)
Supplement: Supplementary file 2 — Supplementary Material 2 [file 18_2024_5377_MOESM2_ESM.docx]

**Supplementary figures**

Figure S1


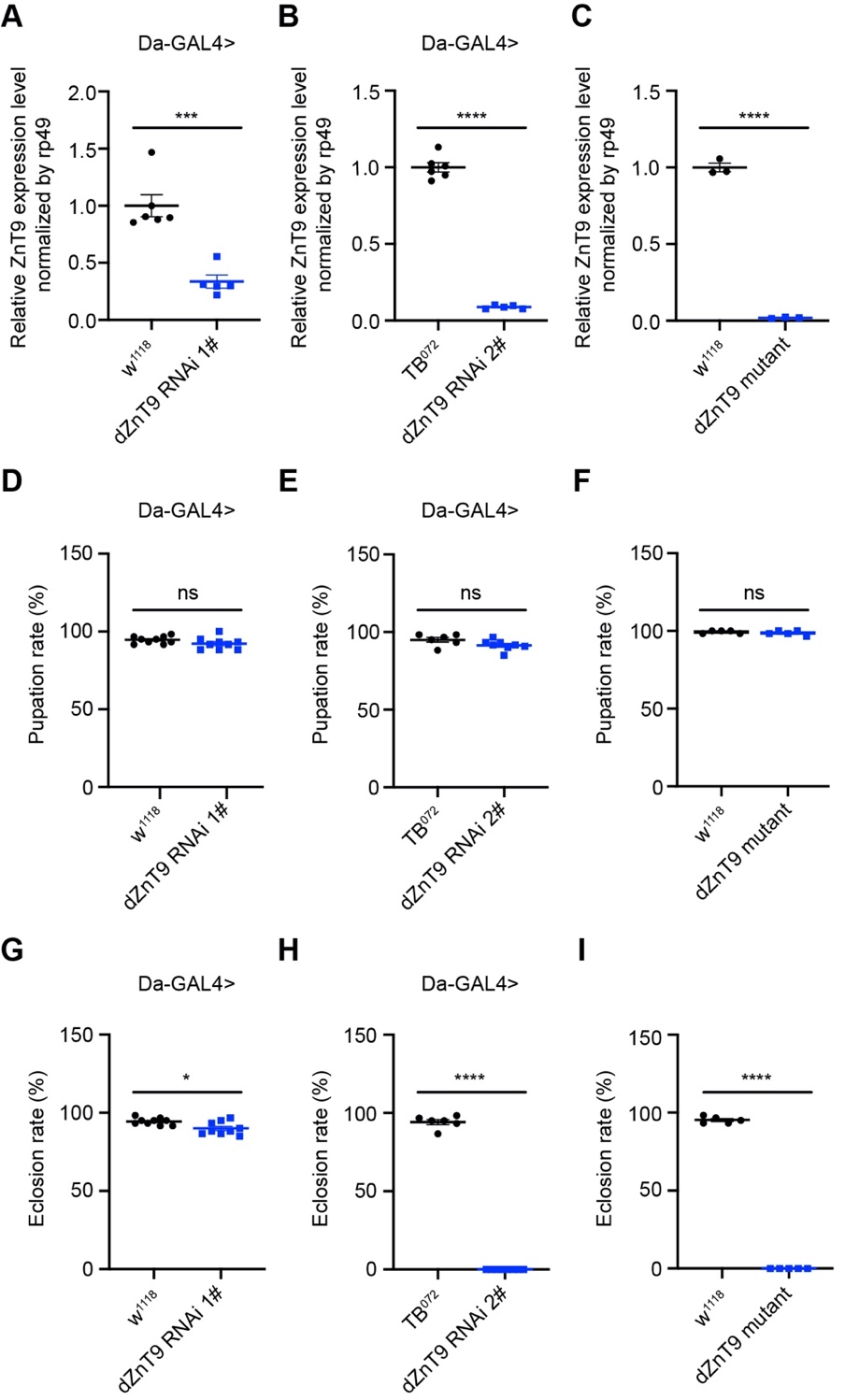


**Fig. S1 *dZnT9* loss decreased eclosion rates.** (A) *dZnT9* expression levels in WT and *dZnT9* RNAi 1# flies (n = 6 and 5 for WT and *dZnT9* RNAi 1#, respectively). (B) *dZnT9* expression levels in WT and *dZnT9* RNAi 2# flies (n = 6 and 5 for WT and *dZnT9* RNAi 2#, respectively). (C) *dZnT9* expression levels in *dZnT9* mutant flies (n = 3 per group). (D) The pupation rate of WT and *dZnT9* RNAi 1# flies (n = 9 per group, each sample includes 60 flies). (E) The pupation rate of WT and *dZnT9* RNAi 2# flies (n = 6 per group, each sample includes 60 flies). (F) The pupation rate of *dZnT9* mutant flies (n = 5 per group, each sample includes 60 flies). (G) The eclosion rate of WT and *dZnT9* RNAi 1# flies (n = 9 per group, each sample includes 60 flies). (H) The eclosion rate of WT and *dZnT9* RNAi 2# flies (n = 6 per group, each sample includes 60 flies). (I) The eclosion rate of *dZnT9* mutant flies (n = 5 per group, each sample includes 60 flies).

Figure S2


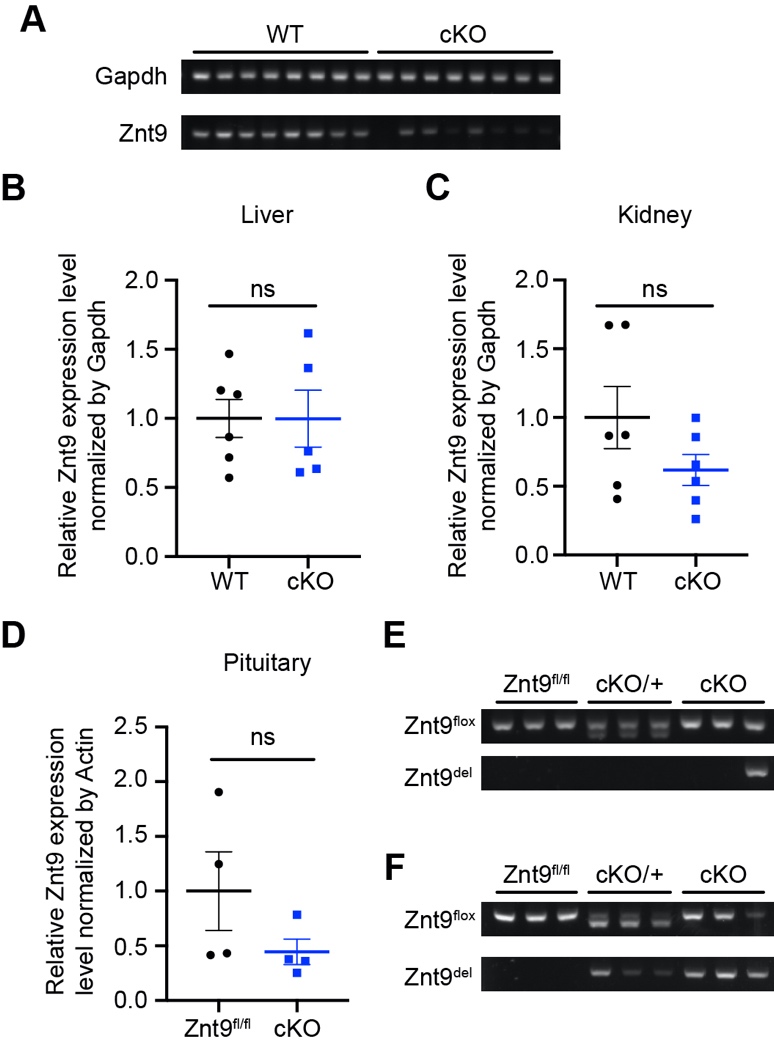


**Fig. S2 *Znt9* expression levels in different tissues of WT and cKO mice.** (A) RT-PCR for *Znt9* in the brain of WT and cKO mice (n = 8 mice per group). (B) qPCR for *Znt9* in the liver of WT and cKO mice (n = 6 and 5 for WT and cKO, respectively). (C) qPCR for *Znt9* in the kidney of WT and cKO mice (n = 6 mice per group). (D) qPCR for *Znt9* in the pituitary of *Znt9^fl/fl^* and cKO mice (n = 4 per group). A sample was pooled from three pituitaries of the same genotype. (E) Genomic PCR for *Znt9* in the pituitary of *Znt9^fl/fl^* , cKO/+ and cKO mice (n = 3 mice per group). (F) Genomic PCR for *Znt9* in the hypothalamus of *Znt9^fl/fl^* , cKO/+ and cKO mice (n = 3 mice per group).

Figure S3


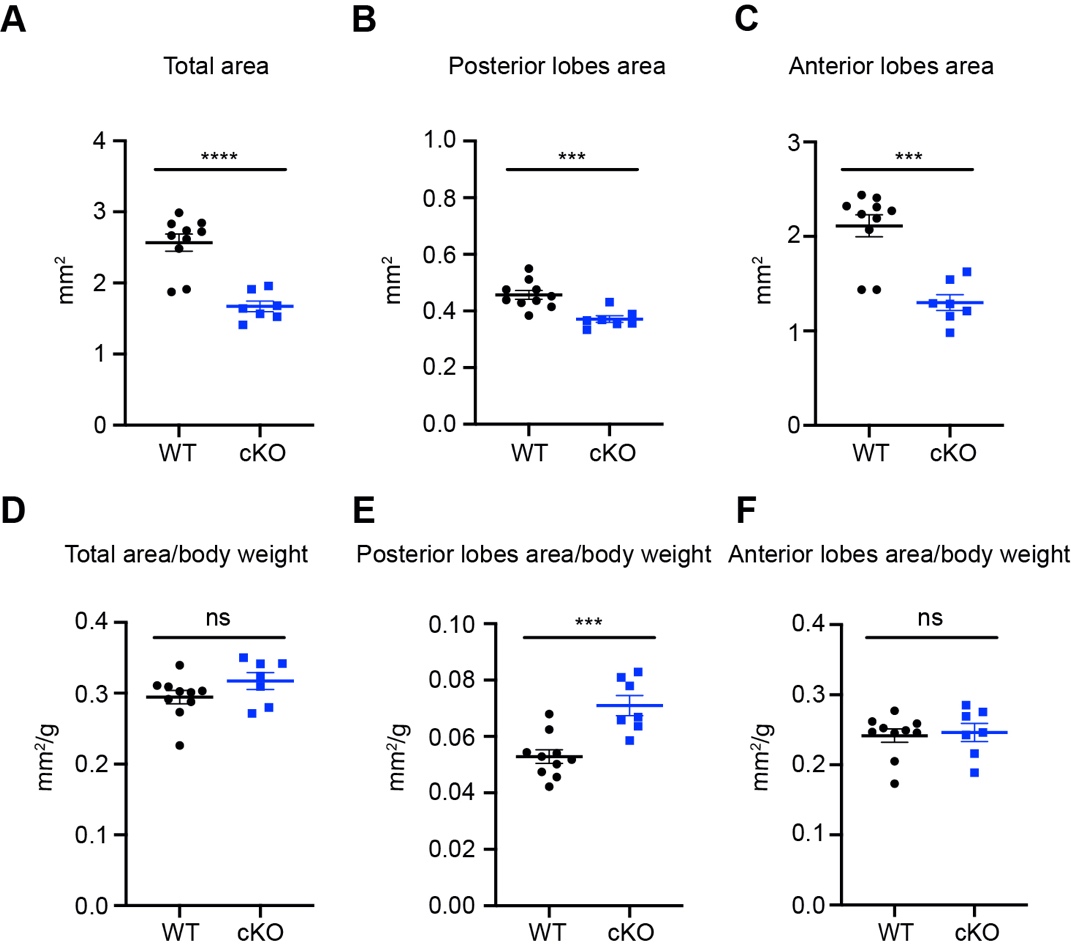


**Fig. S3 Quantification of the pituitary size of *Znt9* cKO mice.** (A-C) Quantification of total area, posterior area and anterior area of WT and cKO pituitaries in Fig. 7A. (D-F) Quantification of the ratio of total area, posterior lobes area, and anterior lobes area of pituitaries to body weight in Fig. 7A.

Figure S4


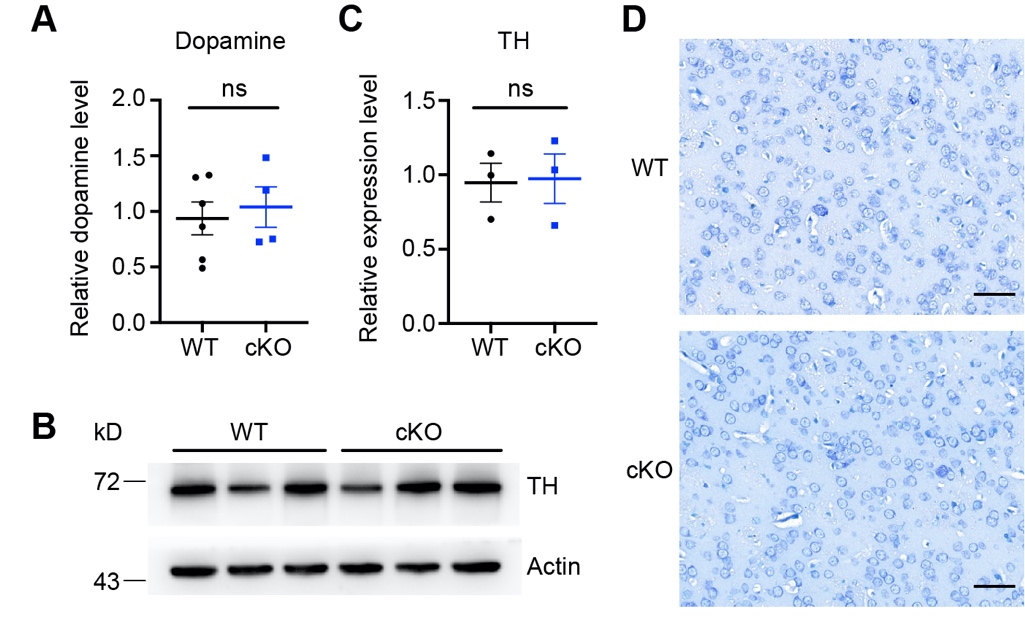


**Fig. S4 The dopamine level in *Znt9* cKO striatum did not show abnormality.** (A) HPLC analysis of dopamine level in striatum from WT and cKO mice (n = 6 and 4 for WT and cKO, respectively). (B) Western blot analysis of substantia nigra using anti-TH antibody (n = 3 mice per group). (C) Quantification of TH expression level in WT and cKO mice in B. (D) Nissl staining of striatum from WT and cKO mice (n = 5 mice per group). Scale bar = 0.05 mm.

Figure S5


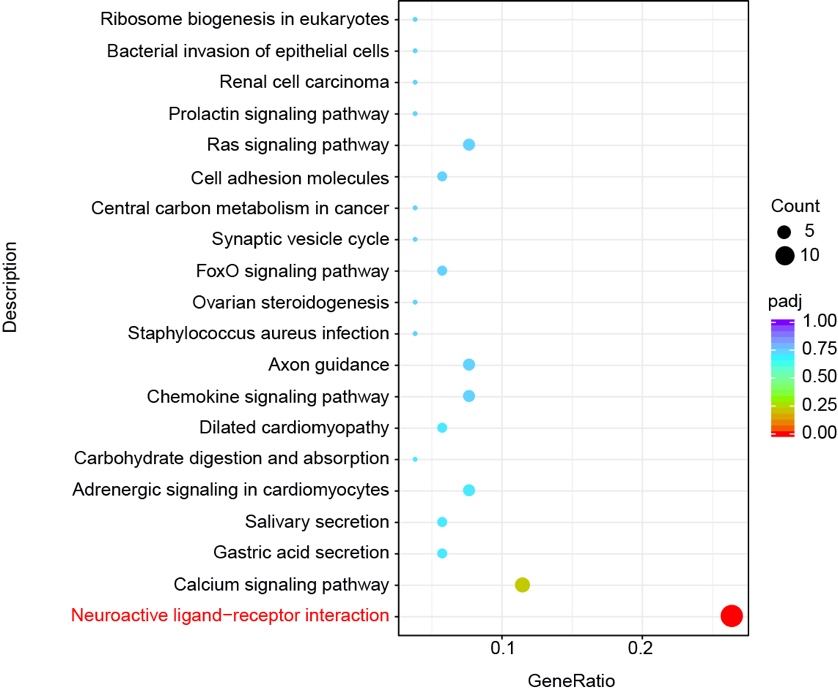


**Fig. S5 Kyoto Encyclopedia of Genes and Genomes (KEGG) analysis of RNA-seq result.** Note that the significant downregulated pathway (padj < 0.05) is neuroactive ligand-receptor interaction which is written in red.

Figure S6


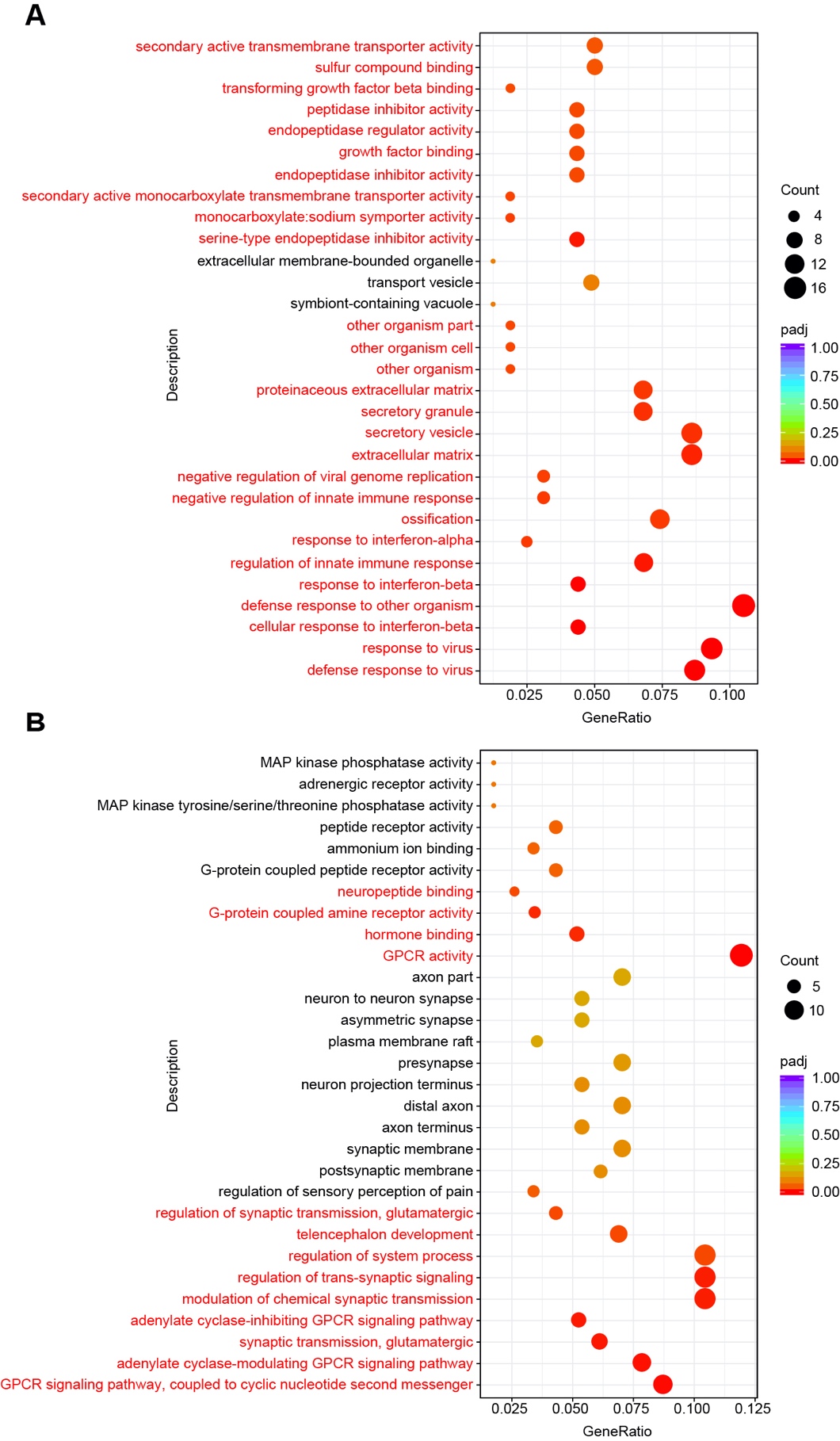


**Fig. S6 Gene Ontology (GO) analysis of RNA-seq result.** The significant upregulated pathway (padj < 0.05) (A) and significant downregulated pathway (padj < 0.05) (B) are written in red.
